# Selective Silencing of Disease-Associated B Lymphocytes from Hashimoto’s Thyroiditis Patients by Chimeric Protein Molecules

**DOI:** 10.3390/ijms232315083

**Published:** 2022-12-01

**Authors:** Nikola Ralchev Ralchev, Aleksandar Mishel Markovski, Inna Angelova Yankova, Iliyan Konstantinov Manoylov, Irini Atanas Doytchinova, Nikolina Mihaylova Mihaylova, Alexander Dimitrov Shinkov, Andrey Ivanov Tchorbanov

**Affiliations:** 1 Laboratory of Experimental Immunology, Institute of Microbiology, Bulgarian Academy of Sciences, 1113 Sofia, Bulgaria; 2 Department of Endocrinology, Medical Faculty, Medical University of Sofia, 1431 Sofia, Bulgaria; 3 Faculty of Pharmacy, Medical University of Sofia, 1000 Sofia, Bulgaria; 4 National Institute of Immunology, 1517 Sofia, Bulgaria

**Keywords:** Hashimoto’s thyroiditis, protein chimeric molecules, autoimmunity

## Abstract

Hashimoto’s thyroiditis is one of the most common endocrine disorders, affecting up to 20% of the adult population. No treatment or prevention exists except hormonal substitution for hypothyroidism. We hypothesize that it may be possible to selectively suppress anti-thyroglobulin (Tg) IgG antibody-producing B lymphocytes from HT patients by a chimeric protein molecule containing a monoclonal antibody specific for the human inhibitory receptor CR1, coupled to peptide epitopes derived from Tg protein. We expect that this treatment will down-regulate B-cell autoreactivity by delivering a strong inhibitory signal. Three peptides—two epitope-predicted ones derived from Tg and another irrelevant peptide—were synthesized and then coupled with monoclonal anti-human CR1 antibody to construct three chimeric molecules. The binding to CD35 on human B cells and the effects of the chimeric constructs on PBMC and TMC from patients with HT were tested using flow cytometry, ELISpot assay, and immunoenzyme methods. We found that after the chemical conjugation, all chimeras retained their receptor-binding capacity, and the Tg epitopes could be recognized by anti-Tg autoantibodies in the patients’ sera. This treatment downregulated B-cell autoreactivity and cell proliferation, inhibited Tg-specific B-cell differentiation to plasmablasts and promoted apoptosis to the targeted cells. The treatment of PBMCs from HT patients with Tg-epitope-carrying chimeric molecules affects the activity of Tg-specific autoreactive B lymphocytes, delivering to them a strong suppressive signal.

## 1. Introduction

Hashimoto’s thyroiditis (HT) is an organ-specific autoimmune disorder, part of the group of heterogeneous autoimmune thyroid diseases (AITDs). It is characterized by abnormal B- and T-cell activation. This pathological reaction is directed against the thyrocites and leads to the destruction of the thyroid and subsequently to hypothyroidism. Together with Graves’ disease (one of the most common causes of chronic hyperthyroidism), these disorders affect up to 15% of adult females, much more commonly than other autoimmune diseases such as type 1 diabetes mellitus and multiple sclerosis [1,2,3]. Most of the cases remain undiagnosed for long periods, but hypothyroidism is associated with various physical and psychological issues. However, no therapeutic approach to prevent HD-induced thyroid destruction has been proven to work and the only treatment is thyroid hormone replacement.

During HT progression, lymphocyte infiltration of overactivated B and T cells in the thyroid gland leads to destruction of thyrocytes by various cell- and antibody-mediated immune mechanisms. The major targets for pathological immune response among the thyroid-specific autoantigens are the thyroglobulin (Tg), the intracellular membrane-bound protein thyroid peroxidase (TPO), and the thyrotropin receptor (TSHR). The breakdown of self-tolerance against the antigens in the thyroid gland is the result from a primary defect in immune recognition and regulation leading to targeting of immune reaction to Tg, TPO, and TSHR [4,5]. Tg, the most abundant protein in the thyroid gland, is produced by thyrocytes and then stored in the follicular lumen. The structural analysis of Tg by use of computer algorithms to explore the probable MHC-binding Tg peptides has found many responding epitopes determining the Tg as a T/B-cell attractant, facilitating their activation with subsequent autoimmunity [6].

The growing importance of HLA class II genes for susceptibility to autoimmune thyroiditis (AITD) has been elucidated in recent years. Some of them, such as HLA-DR3 [7,8], -DR4 [7], and -DR5 [9] are related to AITD in Caucasians, while others (HLA-DQ2 and -DQ8) are present in half of the patients with AITD [10,11].

Autoreactive B and T cells may play diverse roles in the pathology of autoimmunity, but together they form an effector and amplifying pair involved in the development of HT. During HT progression, a diverse array of autoantibodies is generated, those to Tg being frequently the first to be detected. Increased levels of Tg-specific autoantibodies are associated with disease development determining Tg as one of the major autoantigens in HT. Autoantibody production is not the only role of B cells for pathology activation. B lymphocytes are recognized as potential antigen-presenting cells for processing and presentation to T cells [5,12]. Due to the specific role that autoreactive B lymphocytes play in the induction of HT, their selective inhibition is a logical goal in the attempt to suppress the manifestations of the disease.

On the other hand, HT-recognizing T cells also play an important role in the development of autoimmunity. HT-specific CD4+ T cells can bind to epitopes of Tg represented by autoreactive B cells and thus amplify B-cell differentiation to plasmablasts and autoantibody-producing plasma cells. Additionally, cytotoxic CD8+ T cells targeted to Tg can destroy the thyrocytes and the thyroid gland by either granule exocytosis or apoptosis [12,13].

A possible approach for downregulation of B-cell activity is to engage inhibitory B-cell receptors through their ligands or monoclonal antibodies. A potential candidate for this purpose is the human complement receptor (CR1, CD35), which provides a strong inhibitory signal to B lymphocytes [14,15]. We have shown in a previous work how to provide the specificity of B-cell targeting by engineered protein chimeric molecules which crosslink the surface antigen-binding receptors with the inhibitory B-cell receptors [16,17,18]. These engineered molecules contain a monoclonal antibody specific to CD35 conjugated to peptide epitopes derived from basic autoantigens. The chimeras were applied in experimental models of systemic lupus erythematosus and type 1 diabetes mellitus and transduced a strong negative signal to disease-associated B cells through the targeted receptors.

The aim of our study was the selective downmodulation of autoreactive anti-Tg B lymphocytes from HT patients with an engineered protein molecule consisting of an anti-CR1 monoclonal antibody coupled to peptide epitopes derived from human Tg.

## 2. Results

### 2.1. In Silico Tg Peptide Binding Prediction

**Figure 1:**
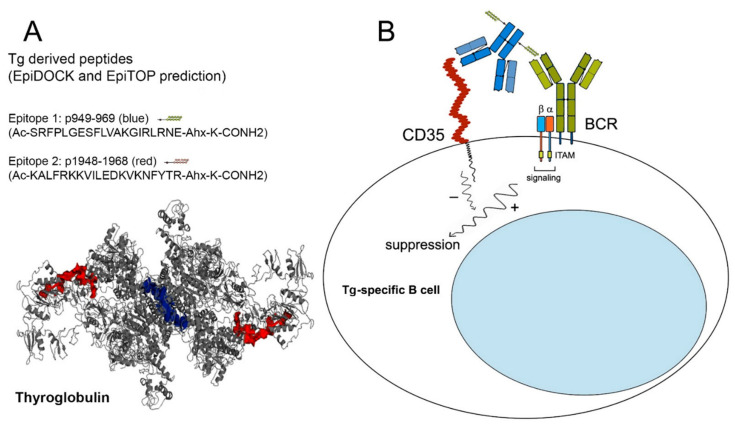
The generated Tg chimeras suppress selectively disease-associated Tg-specific B cells by crosslinking the BCR and CD35 on the cell surface. (**A**) Structure of human Tg dimer (pdb code: 6SCJ). Epitope 1 (p949-969) is shown in blue. Epitope 2 (p1948-1968) is shown in red. The image was generated by PyMOL Molecular Graphics System, version 2.5.0. (**B**) The co-crosslinking of Tg-specific BCR and the inhibitory CR1 receptor on pathological autoreactive B lymphocytes generates negative signal transduction for B-cell activation.

Using EpiTOP and EpiDOCK servers, we predicted several Tg peptides binding to AITD-susceptible HLA alleles as described previously [19]. The resulting 10 predicted best binders for each allele were mapped on the Tg sequence (UniProtKB: P01266) and 54 peptide fragments containing 1–15 binders were identified. The peptide fragments consist of overlapping or nonoverlapping binding cores and four flanking residues (two of each end). Finally, seven fragments containing peptides associating to at least five of eight alleles were selected. Further, the binder identities in human and mouse Tgs were compared and five peptide fragments (p470, p949, p1948, p2348, and p2583) showing 75% or higher homology were found among them (Table 1). Two peptides (p949 and p1948) were selected for analysis and chimera construction. The peptide p949 (20 residues) had eight predicted binders to five DR and one DQ alleles and showed 88% homology between the human and the mouse Tgs, while the peptide p1948 had six predicted binders to six alleles with 75% homology. The last one carried known T-cell epitope ILEDKVKNF, relevant to AITD [20]. The location of the two peptides could be seen on the dimer Tg structure (Figure 1A).

### 2.2. Assembling and Functional Assay of the Protein Chimeric Molecules

The newly-engineered protein chimeras (Tg chimeras 1/2 and Control chimera) were constructed by covalent joining of multi copies of the synthetic peptides p949-969, p1948-1968 or Control peptide to the monoclonal anti-human CD35 antibody (Figure 1B).

**Figure 2:**
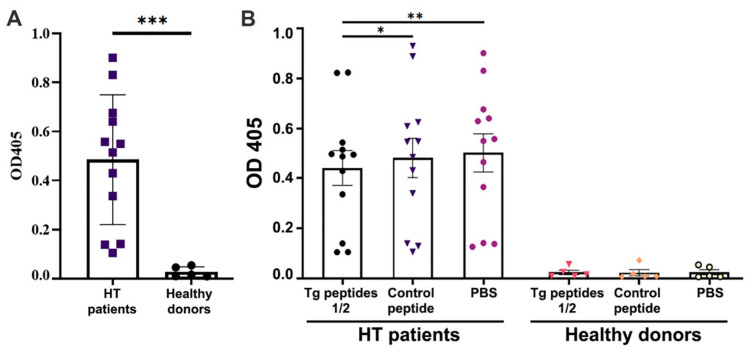
The thyroid gland lysate and Tg peptide epitopes are recognized by anti-Tg IgG antibodies from HT patients. (**A**) Autoantibodies in sera from HT patients recognized the thyroid gland lysate by ELISA. (**B**) ELISA assay for peptide epitopes recognition by antibodies in the individual sera from HT patients (left) and healthy donors (right). The sera were preincubated with Tg1/Tg2 peptides mix or with Control peptide and later incubated in 96-well plates loaded with thyroid gland lysate. All assays were performed in triplicate and the average values were used for analysis. Mean ± SD values were calculated for each group; p values were calculated (*n* = 12 for HT patients and *n* = 5 for healthy donors) (* *p* < 0.05, ** *p* < 0.01, *** *p* < 0.001) in comparison to sera preincubated with PBS only.

First, a thyroid tissue lysate from subjects without HT was prepared. The recognition of antigens in the lysate by the antibodies in the patients’ sera was tested using the sera of patients with HT (*n* = 12) and healthy donors (*n* = 5) by ELISA (Figure 2A). The results showed that the sera of patients with HT had a high titer of antibodies against the lysate antigens, while those isolated from the blood of healthy donors did not show the presence of antibodies against the lysate.

In the next experiment, we checked whether the predicted epitopes in Tg1 and Tg2 peptides were recognized by antibodies in patients’ sera. For this purpose, we preincubated the sera from HT patients and healthy donors with a mix of Tg1 and 2 as well as with the control peptide or with PBS (Figure 2B). The inhibition of antibody binding to lysate showed that peptides Tg1 and 2 bound anti-Tg antibodies from the HT patients’ sera to a much greater extent than the control peptide. In healthy donors, binding was negligible and was not inhibited.

**Figure 3:**
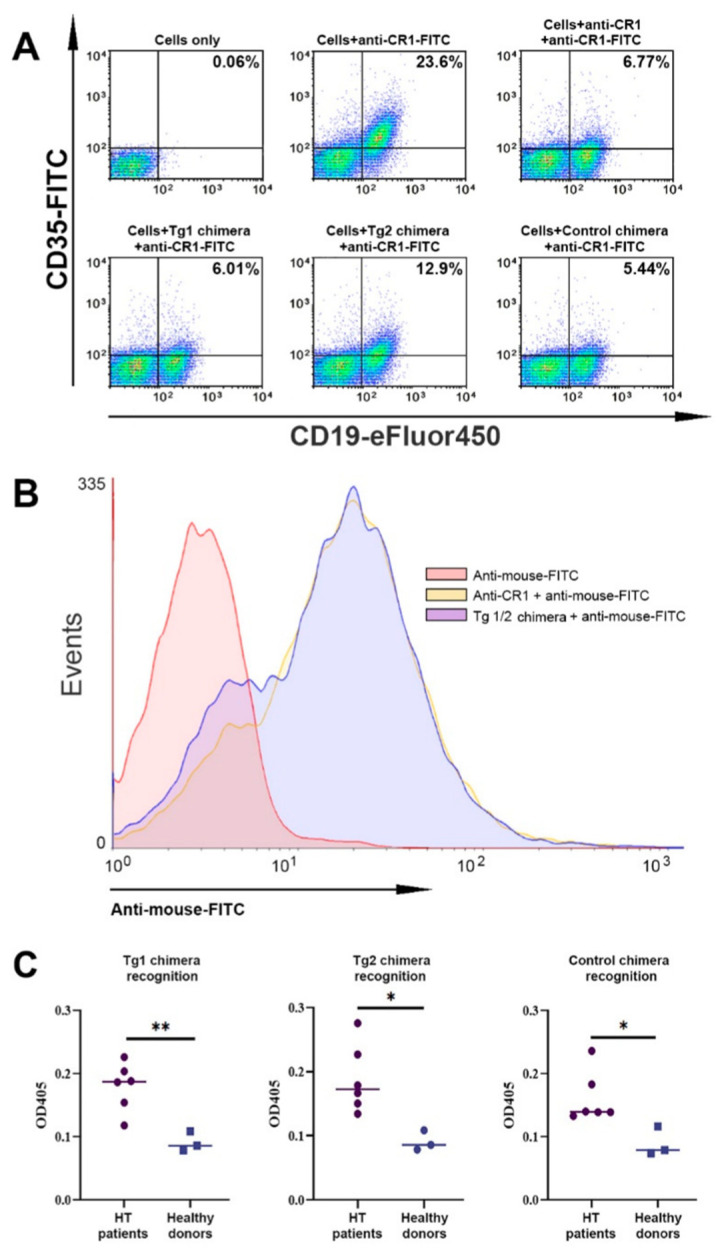
Tg 1/2 chimeras bind to CD35 expressed on B cells isolated from HT patients and are recognized by anti-Tg IgG antibodies. (**A**) A competition binding assay for CD35 was performed by flow cytometry. Isolated PBMCs from HT patients were incubated with the Tg1 chimera, or with the Tg2 chimera, or with the Control chimera, or with unconjugated 3D9 MoAb, or PBS alone, followed by incubation with anti-human CD19-eFlour450/CD3-PeCy7 antibodies together with FITC-conjugated anti-human CD35. (**B**) The same PBMCs were incubated with the Tg1/2 chimeras, or with unconjugated 3D9 MoAb, or with PBS alone, followed by incubation with FITC-conjugated anti-mouse IgG antibody together with CD19-eFlour450/CD3-PeCy7 antibodies. Gated CD19+ or CD3+ lymphocytes were analyzed by flow cytometry. Data are representative of at least 6 independent experiments. (**C**) The Tg1 and Tg2 epitopes retain their capability to be recognized by anti-Tg IgG antibodies. Tg 1/2 peptide recognition was performed by ELISA assay. The Tg1 chimera (left), Tg2 chimera (middle), and Control chimera (right) were loaded on the immune plate and incubated with diluted sera from HT patients or healthy subjects. All samples were triplicated and average values were used for analysis. Mean ± SD values were calculated for each group; *p*-values were calculated (* *p* < 0.05, ** *p* < 0.01). Data are representative of 4 independent experiments.

During the process of conjugation, several synthetic peptides could interact with their own reactive H_2_N group from the C-end Ahx linker with the space-available free carboxyl groups on the immunoglobulin backbone. The carboxyl groups within the antigen-binding sites are well encrypted, while the other ones nearby could affect the BCR activity after the conjugation. To prove that this did not happen, we tested the binding capacity of chimeric molecules to CD35 expressed on human B and T cells isolated from HT patients against a FITC (flourescein isothiocyanate)-conjugated antibody with the same specificity. Competitive fluorescence-activated cell sorting (FACS) analysis showed that Tg chimeras 1 and 2 as well as the control chimera retained their antigen-binding activity and inhibited the binding of FITC-labeled anti-CD35 antibody to the CD19+ cells from patients with HT (Figure 3A). Such inhibition was not observed in CD3+ cells, indicating preferential binding of chimeras to B lymphocytes. In addition, we performed an alternative experiment in which we first incubated peripheral blood mononuclear cells (PBMCs) from HT patients with unconjugated anti-CR1 antibody, with Tg 1/2 chimeras, or with PBS with subsequent incubation with anti-mouse IgG antibody FITC, which demonstrated effective binding of the chimeric molecules to the target receptor (Figure 3B).

Further on, we tested the epitopes’ availability for antibody recognition as part of the chimeric molecules. The Tg1, Tg2, or Control chimeric molecules were subjected to ELISA for interaction with the anti-Tg antibodies in sera from HT patients (*n* = 6) or healthy volunteers (*n* = 3). The anti-Tg IgG antibodies in the HT patients’ sera recognized the peptides in Tg chimeras 1 and 2 more extensively than those of healthy donors, and recognized structures in the Control chimera only partially (Figure 3C).

### 2.3. Tg 1/2 Chimeras Suppress Cell Proliferation In Vitro

**Figure 4:**
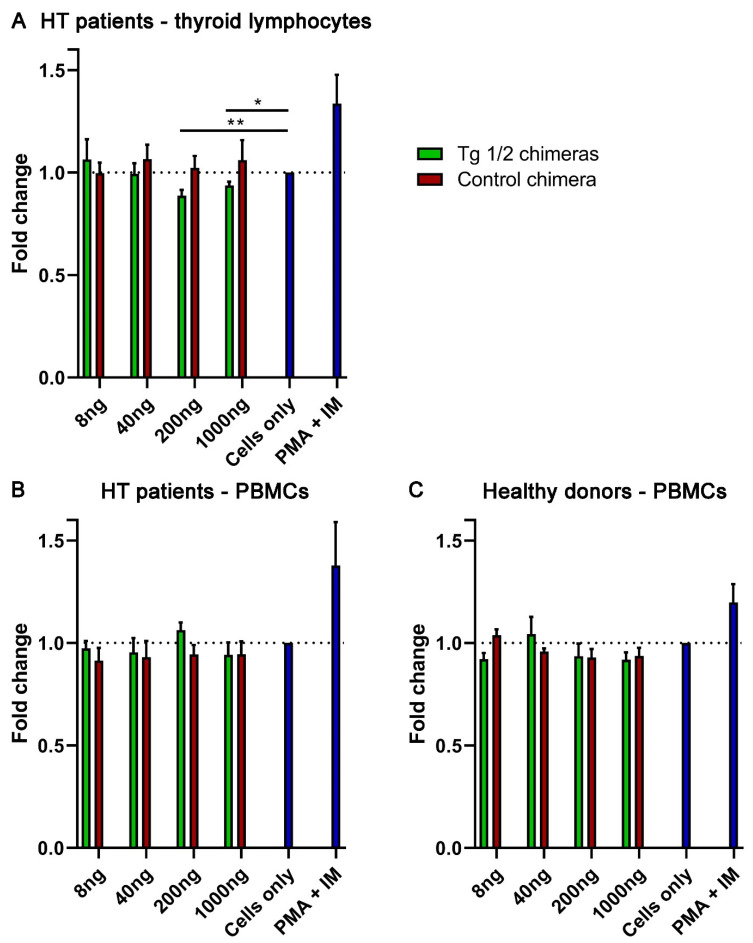
The Tg1/2 chimeras inhibit in vitro the proliferation of TMCs. TMCs (**A**) and PBMCs (**B**) from HT patients or healthy donors (**C**) were co-cultured with rising concentrations of Tg 1/2 chimeras or Control chimera and the cell proliferation was evaluated by MTT assay compared to the untreated cells. All samples were triplicated and average values were used for analysis. Mean ± SD values of fold change were calculated for each group; *p*-values were calculated (* *p* < 0.05, ** *p* < 0.01). Data are representative of 4 independent experiments.

To explore the effect of Tg 1/2 chimeric molecules on intrathyroidal mononuclear cells (TMCs) or PBMCs isolated from HT patients and healthy donors (*n* = 3–5) we investigated several cell activities and functions. Using a colorimetric MTT (3-[4,5-dimethylthiazol-2-yl]-2,5- diphenyltetrazolium bromide) assay which is indicative of the mitochondrial activity of the cells, we followed the suppression of cell proliferation after incubation with different concentrations of chimeric molecules without stimulation and treatment with other stimuli. The in vitro treatment of TMCs with 200 and 1000 ng/mL Tg 1/2 chimeras suppressed significantly cell proliferation in dose-dependent manner compared to untreated cells, while the control chimera did not affect the cell proliferation (Figure 4A). No significant differences were found after chimera treatments using PBMCs isolated from HT patients (Figure 4B) and healthy donors (Figure 4C).

### 2.4. The Tg1/2 Chimeras Suppressed the Tg-Specific B-Cell Differentiation to Anti-Tg Antibody-Secreting Plasmacytes

**Figure 5:**
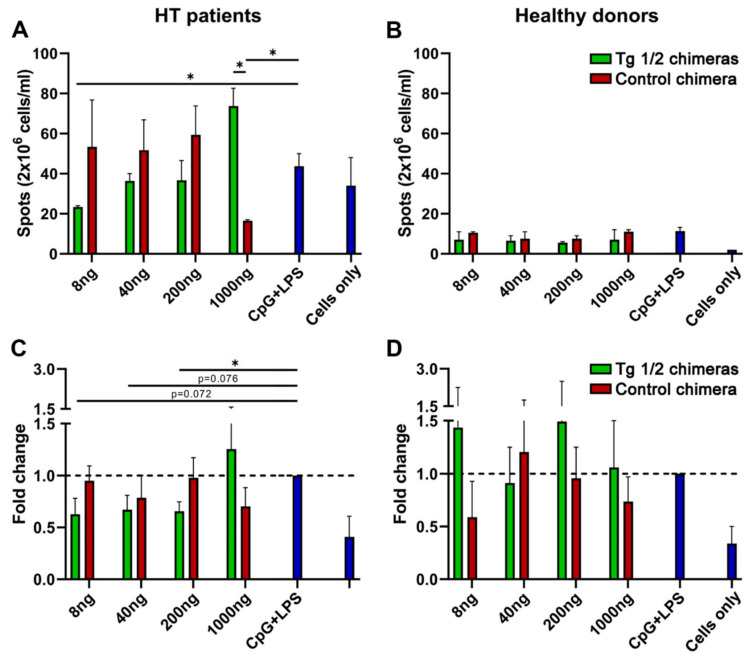
The Tg1/2 chimeras suppressed the Tg-specific B-cell differentiation to anti-Tg antibody-secreting plasmacytes. PBMCs from HT patients (**A**,**C**) and healthy donors (**B**,**D**) were cultured with the Tg 1/2 chimeras, or with the Control chimera, or cultured in medium only for 24 h followed by 3 days’ incubation with CpG + LPS or medium only. The number of spots in the test-wells was compared to untreated cells (CpG + LPS control) and determined by ELISpot assay. Results from representative HT patient (**A**) and healthy donor (**B**) are shown. Summarized results for the calculated fold change of 3 independent experiments are presented (**C**,**D**). All samples were triplicated and average values were used for analysis. Mean ± SD values were calculated for each group; *p*-values were calculated (* *p* < 0.05).

To test whether chimeras affect the ability of B cells to secrete antibodies, we performed an ELISpot assay. Treatment of the cells from HT patients with low concentrations of Tg1/2 chimeric molecules achieved suppression of the Tg-specific plasma cells compared to PBMCs treated with Control chimera and CpG + LPS control cells (significance at 8 ng/mL, Figure 5, left panel). Non-specific suppression with Control chimera was observed after treatment with 1000 ng/mL.

The very low number of Tg-positive plasma cells among the PBMCs isolated from healthy donors were not affected by the same treatment (Figure 5, right panel).

### 2.5. The Chimeric Molecules Increased the B-Cell Apoptosis

**Figure 6:**
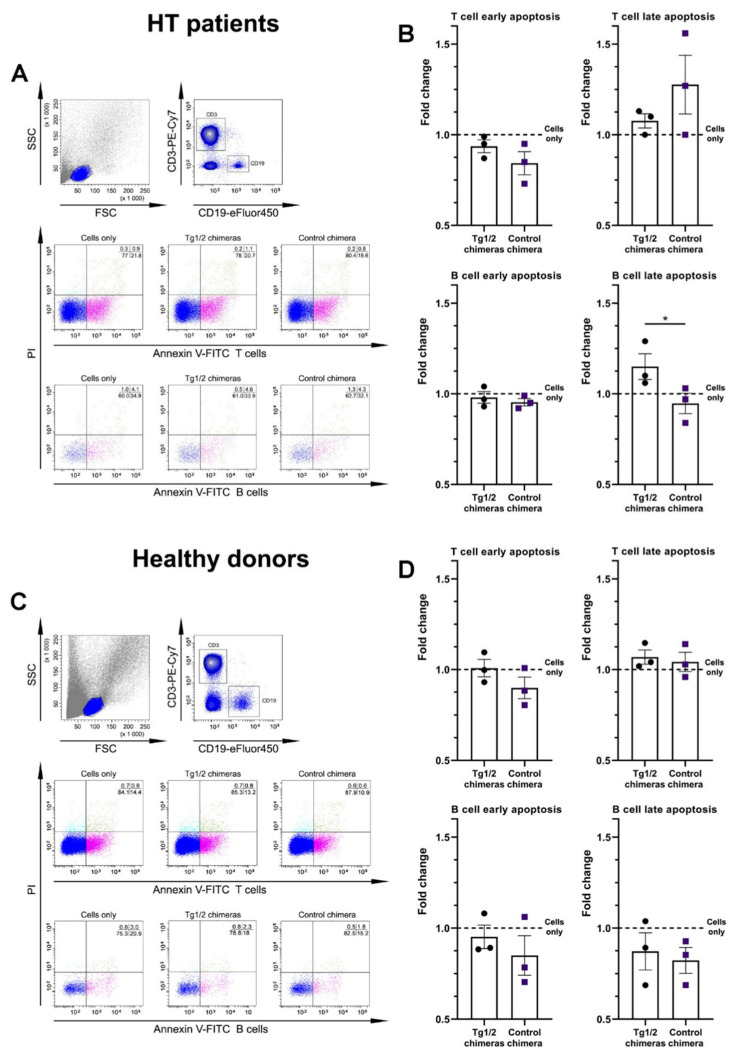
Tg 1/2 chimeras increase B-cell apoptosis. Isolated PBMCs from HT patients and healthy donors were co-cultured with 8 ng/mL Tg1/2 chimeras, or with Control chimera, or cultured in medium alone for 2 days, after which the cells were stained by Annexin V-FITC/PI and analyzed by FACS. The percentage positive cells within gated T or B lymphocytes are presented as plot graphs (**A**,**C**), and one typical experiment from the three performed is shown. The extracted results for fold change of the early or late apoptosis of CD19+ and CD3+ lymphocytes are presented graphically (**B**,**D**), and *Cells only* control is visualized as a baseline. The data are represented as mean ± SD (*n* = 3), (* *p* < 0.05).

The pro-apoptotic effect of Tg1/2 chimeras on B and T lymphocytes was investigated using isolated PBMCs from HT patients and healthy donors. The cells without stimulation were co-cultured with the Tg1/2 chimeras, the Control chimera, or cultured with medium only. The CD3 or CD19-gated lymphocytes were analyzed by flow cytometry for the surface expression of phosphatidylserine and DNA staining with PI (propidium iodide). The treatment of PBMCs isolated from HT patients with 8 ng/mL Tg 1/2 chimeras resulted in a significantly increased late apoptosis within CD19+ cells (Figure 6A,B). The early apoptosis was nonsignificantly decreased after the respective chimera treatments.

The apoptosis level of untreated B or T cells from healthy individuals was weak compared to the apoptosis of lymphocytes isolated from HT patients, and the treatment either with Tg1/2 chimeras or with Control chimera did not have significant pro-apoptotic effects within gated T and B cells.

## 3. Discussion

The disease pathogenesis and secondary markers of tissue damage have been elucidated in most autoimmune diseases, but the main question of which specific antigens are the targets that initiate the autoimmune process still exist. Under normal physiological conditions, small disruptions of the follicles in the thyroid gland result in increased Tg leakage and release into the circulation along with T3 and T4. The Tg molecule exhibits great variability due to the large number of point mutations that do not affect function but might increase Tg immunogenicity. Several pathogenic Tg epitopes have been identified using computer algorithms to predict MHC-binding Tg peptides or elution of human MHC-bound peptides [5,6].

B-cell epitopes are basically located away from the cysteine-rich regions of the tandem sequence repetition suggesting the lapse of normal processing and presentation of these peptides to the immune system and involving of additional mechanisms for cryptic epitope presentation. A number of T-cell immunopathogenic nondominant epitope-bearing peptides have also been identified. Most of them overlap the HT-associated pathological B-cell epitopes situated within the acetylcholinesterase-homologous domain of Tg, while the other T-cell epitopes are within cysteine-rich repeats. The features of these epitopes suggest that HT development might be associated with the unmasking of cryptic epitopes, and whose antigen presentation would break the tolerance to Tg [21]. Similar overlapping of T-cell epitopes within the frame of B-cell epitopes and presentation by autoreactive B cells resulted in blocking or inhibition of antigen-presentation to the respective T-cell clones in a model of human diabetes mellitus type 1 [18,22].

Using the platforms EpiTOP and EpiDOCK, we predicted six T-cell HLA class II protein binding peptides in the sequence of human Tg with possible susceptibility to AITD [19]. Two of them—p949—Tg1 (eight DR3, DR4, DR5, DQ8 binders) and p1948—Tg2 (six DR4, DR5, DQ2 binders) were selected in the present study for specific targeting to Tg-recognizing B cells. Both peptides were recognized by the sera from HT patients and inhibited the interaction of anti-Tg IgG antibodies from the same sera with thyroid antigens. Indeed, using two Tg peptide epitopes for sera preincubation made it possible to block only the two respective antibody clones, while the real number of potential anti-Tg antibodies recognizing various epitopes is much higher. For this reason, we cannot expect the total suppression of anti-Tg binding.

The lack of effective treatment of HT draws attention to the regulation of the pathological immune response. The activity of B cells involved in the autoimmune process may be affected by binding specific cellular surface molecules. The monoclonal antibody rituximab recognizes CD20 molecules on B cells and contributes to their depletion by complement and Fc receptor-mediated lysis. It has been used with beneficial effect for the treatment of autoimmune diseases such as rheumatoid arthritis, multiple sclerosis, and SL, but this therapy is not successful in autoimmune thyroiditis [23,24,25,26,27]. Rituximab is only used to treat selected patients with thyroid-associated orbitopathy, suggesting participation of antibody presentation by B cells [28]. Moreover, the application of rituximab is limited due to the lack of expression on B-cell precursors and long-term plasma cells [29].

Another possible option is B-cell suppression by either anti-CD22 antibody (epratuzumab) or antibody blocking of the Lympho-Stat-B, B-cell Activating Factor (BAFF) or a proliferation-inducing ligand (APRIL), which are under investigation for patients with rheumatoid arthritis and lupus but are without application to HT yet [30,31,32,33]. These nonspecific approaches for total B-cell depletion may result in partial immune deficiency and subsequent emergence of new-onset autoimmune disorders. To increase the specificity of B-cell downregulation, a crosslinking of surface immunoglobulin receptors (BCR) with inhibitory B-cell receptors could be accomplished using artificial chimeric molecules.

We have shown earlier the selective suppression of pathologic human B cells in other autoimmune diseases such as systemic lupus (SLE) and type 1 diabetes mellitus (T1DM) by protein chimeric molecules. These engineered antibodies consist of multiple peptide epitopes recognized in SLE and T1DM and coupled to an anti-human CD35 monoclonal antibody that crosslinks their inhibitory CD35 with BCRs. The administration of SLE- or T1DM-chimeric molecules resulted in a significant reduction in anti-dsDNA or anti-GAD65 IgG antibody-producing plasma cells, inhibition of disease-associated cell proliferation, induction of B- and T-lymphocyte apoptosis as well as reduction in proteinuria and glomerular deposition of human IgG immune complexes in humanized immunodeficient SCID mice reconstituted with PBMCs from SLE patients [16,17,18].

In the present study, we explored the ability of two newly generated protein chimeras consisting of Tg-derived peptide epitopes (Tg1 and Tg2) conjugated to a human anti-CD35 monoclonal antibody to suppress Tg-specific HT-associated B cells producing anti-Tg antibodies.

We needed to prove the functional activity of both elements of the designed chimeric constructs by FACS and ELISA since the antibody affinity or epitope availability could be affected as a result of peptide conjugation. We expected specific binding preferences and higher recognition avidity of the constructed chimeric molecules to Tg-specific B cells due to the additive affinity to both cell receptors (CD35 and BCR) rather than single binding with any protein chimeric element. Indeed, the Tg1/2 chimeras were able to crosslink both targeting receptors and bound preferentially to Tg-specific CD19+ B cells isolated from HT patients. The inhibition effect to CD35-FITC binding or positive cell binding (as % bound cells) was the same as unconjugated anti-CD35 antibody. No specific binding was observed within CD3+ cells either from HT patients or healthy donors.

Further on, we exploited the accessibility of the Tg-derived peptides comprising T-cell epitopes as part of the engineered chimeric molecules for interaction with anti-Tg antibodies. As expected, both chimeric molecules interacted primarily with the HT patients’ sera and not the healthy donors’ sera. The control peptide as part of the Control chimera was recognized with lower intensity from either IgG antibodies in the sera of patients or healthy donors. This can be explained by the generation of a large set of poly-specific antibodies characteristic of autoimmune diseases.

The coupled action of disease-associated B and T lymphocytes could be disturbed by the Tg1/2 chimeras through several mechanisms: interruption of B/T-cell communication with blocking of antigen presentation from Tg-specific B lymphocytes; inhibition of B-cell differentiation to plasmacytes; or restriction of antibody production from the plasma cells. In silico prediction of these potential epitopes and the generation of bivalent engineered molecules allows for a selective approach for the elimination of the persisting Tg-specific B lymphocytes without nonspecific suppression of the remaining B-cell pool. In fact, the in vitro treatment of TMCs isolated from HT patients with Tg 1/2 chimeras significantly suppressed the total cell proliferation in a dose-dependent manner with significance in higher doses, while the Control chimera did not exhibit any inhibitory effect. In contrast, the same treatment suppressed significantly the number of IgG anti-Tg antibody-producing plasma cells with lowest Tg 1/2 chimera concentration, while a nonspecific suppressive effect was found using the highest concentration of the Control chimera. The silencing of pathological Tg-specific B cells by the Tg 1/2 chimeric antibodies is expected to bring down the activation state as well as the numbers of respective Tg-specific T cells.

It was very important to find the minimal treatment dose affecting all potential targets—Tg-specific autoreactive B cells. Using the minimal dose of Tg1/2 chimera, enough for bi-specific engagement of pathological Tg-specific B cells only, we can observe the suppressive effect on them. An increase in Tg1/2 chimera quantity with excess over the Tg-specific B cells allows nonspecific chimera binding to all CR1-expressing cells, resulting in Tg1/2 epitope presentation and opposite disease-stimulating effects.

No effect was observed in MTT and ELISpot assays after the exposure of PBMCs from healthy donors to the Tg 1/2 or Control chimeric molecules.

A number of B-cell abnormalities such as overactivation and lymphopenia are found in systemic lupus [34] and T1DM [35]. The phenotype analysis of Tg-specific B cells in the peripheral blood of AITD patients showed reduced numbers of anergic Tg-specific B cells in blood and increased expression of CD86 compared to healthy controls, suggesting over-activation and increased capacity for antigen-presentation to Tg-specific autoreactive T cells [36]. The co-culturing of patients’ PBMCs with Tg1/2 chimeras increased significantly the late apoptosis levels (Annexin V/PI-double positive) of B cells, while the Control chimera had no effect on the same cells. The opposite effect was observed on the early apoptosis rate, suggesting a specific interaction of Tg-specific B cells.

Tg 1/2 or Control chimeras did not bind to CD3+ T lymphocytes, but a nonsignificantly increased percentage of late apoptosis was found within CD3+ cells after exposure of PBMCs from HT patients to chimeric molecules. The apoptosis of Tg 1/2 chimera-treated patients’ T cells might be explained by an inhibited antigen presentation by the suppressed Tg-specific B cells, while the effect on the same cells co-cultured with the Control chimera could be explained by a nonspecific binding to various overactivated B cells under in vitro conditions and suppressed signals to T cells.

The significant difference observed by us at low concentrations on the studied chimeras may be indicative of the specificity of the chimeras and the efficient receptor co-ligation. At low concentrations, the chimeric constructs bind more and predominantly to autoreactive B cells. With an excess of chimeras at higher concentrations, specificity and selectivity of binding was probably lost and the probability of simultaneous engagement of multiple CR1 on the same B cell and suppression of its activity increased. Neither B nor T cells from healthy donors were significantly affected by chimera treatment.

This study has several limitations. We cannot expect to suppress the whole spectrum of autoreactive lymphocytes during HT progression with engineered molecules comprising only two Tg epitopes, even with the antigen-spreading phenomenon, but the precise suppression of B cells with these epitope specificities provides an opportunity for future multi-epitope delivery. We can expect some weak nonsignificant inhibition in the control samples. The reason for this result is that in both Tg1/2 chimeras as well as in the Control chimera, the anti-CR1 antibody can bind any CR1-positive cell. The presence of Tg-specific autoreactive B cells would engage the Tg1/2 chimeras for both active parts and crosslink CR1 and BCR, providing the specificity of binding.

Another issue that cannot be addressed by the study design is the frequency of point mutations in the Tg molecule and the nonpredictability of epitope variability in the particular patient. Nevertheless, moving to in vivo experiments, we might enhance the effectiveness of targeting the specific Tg-recognizing B cells by Tg1/2 chimeras in real conditions through their bivalency. Furthermore, other thyroid antigens such as thyroid peroxidase, for instance, are involved in thyroid autoimmunity and should be addressed as well.

## 4. Materials and Methods

### 4.1. Monoclonal Antibodies

Mouse anti-human CD35 monoclonal IgG1 antibody (clone 3D9) was prepared as described [17]. FITC-conjugated mouse IgG1 Isotype Control (eBioscience, Frankfurt, Germany) was used for FACS experiments. Anti-human CD35-FITC (clone 3D9, kindly provided by Dr. J. Prechl, Diagnosticum zrt, Budapest, Hungary), anti-human CD19-eFlour450, CD3-PeCy5, CD3-PeCy7 (eBioscience), and anti-mouse IgG-FITC (Invitrogen, Waltham, Massachusetts, USA) antibodies were used for FACS experiments. Mouse IgG1 kappa-eFluor 450 and IgG1 kappa-PE-Cy7 Isotype Controls (eBioscience) were used also for FACS experiments.

### 4.2. Prediction of Peptide Binding to MHC

The servers EpiTOP and EpiDOCK were used for prediction of the Tg peptides binding to HLA-DR3 (DRB1*03:01), HLA-DR4 (DRB1*04:01, DRB1*04:04 and DRB1*04:05), HLA-DR5 (DRB1*11:01 and DRB1*12:01), HLA-DQ2 (DQA1*05:01/DQB1*02:01), and HLA-DQ8 (DQA1*03:01/DQB1*03:02) as described [19]. The proteochemometrics-based peptide binding prediction server EpiTOP [37,38] could predict binding to 24 most frequent human HLA-DR, -DQ, and -DP alleles [39] and is freely accessible at http://www.ddg-pharmfac.net/EpiTOP3 (22 March 2020). The resultant affinity is presented as pIC_50_ (−logIC_50_), wherein the IC50 is the concentration that inhibits 50% of the binding of a radiolabeled standard peptide to MHC molecules.

Another server for peptide binding prediction, EpiDOCK, predicts binding to 23 most frequent HLA-DR, -DQ, and -DP alleles based on quantitative matrices derived by molecular docking of peptide combinatorial libraries on HLA class II proteins [40]. The predicted affinity is evaluated as a score assessing the free energy of binding. Both servers predict the peptide binding core consisted of nine successive amino acid residues.

### 4.3. HT Patients and Healthy Donors

#### Blood and Thyroid Tissue Samples

Patients diagnosed with HT (*n* = 14) with high titers of anti-Tg and anti-TPO IgG antibodies, typical ultrasound features, and hypothyroidism were included in the study (female to male ratio 13:1; age range 25–60). The control samples were obtained from five age- and sex-matched healthy blood donors. The study (grant KP-06-H33/15/2019 from the National Science Fund, Bulgaria) was approved by the Local institutional ethics committee at USBALE “Acad. Ivan Penchev” (Standpoint 10A, Protocol 1/11 March 2020) and all subjects signed an informed consent.

Venous blood was collected from HT patients and healthy individuals in sterile blood tubes (BD Vacutainer, Franklin Lakes, NJ, USA). Serum samples of 5 mL were collected from each subject using serum separator tubes (Vacutainer BD-Plymouth.PL67BP.UK, 5 mL) and stored at −80 °C.

Purified PBMCs were obtained from heparinized venous blood of both HT patients and healthy volunteers by Histopaque (PAN Biotech, Aidenbach, Germany) gradient separation at 800× *g* for 20 min at 20 °C and were then cultured in Roswell Park Memorial Institute (RPMI) 1640 medium (Sigma-Aldrich) supplemented with 10% FCS, NaHCO_3_, antibiotics, and 4 mM L-glutamine at 37 °C in 5% CO_2_.

TMCs from HT patients undergoing thyroidectomy were isolated by grinding the gland fragments obtained during surgery (*n* = 4) through a sterile steel mesh and 70 µm cell strainers and then following the gradient separation protocol for PBMCs.

### 4.4. Thyroid Gland Lysate Preparation

Human thyroid tissue samples 1–3 cm^3^ in volume were harvested during thyroidectomy from subjects without HT. The samples were placed immediately in sterile media and transported to the laboratory for processing. The freshly obtained human thyroid gland samples were cut into pieces, which were placed in liquid nitrogen. Later, the tissue was homogenized by mechanical disruption on ice in 500–1000 µL PBS with a 4 mM protease inhibitor (PefaBloc, Roche, Basel, Switzerland), and 1 mM EDTA (Sigma-Aldrich, Taufkirchen, Germany). Tissue extracts were centrifuged at 17,000× *g* for 5 min at 4 °C and the supernatant was measured spectrophotometrically at 280 nm.

### 4.5. Assembling the Chimeric Molecules Containing Tg Epitopes and Anti-CR1 Antibody

Two peptides from the human Tg were selected for chimera construction: p949-969 (SRFPLGESFLVAKGIRLRNE) and p1948-1968 (KALFRKKVILEDKVKNFYTR). The peptides were synthesized with >98% purity (Caslo Laboratory, Lyngby, Denmark), and during the process a lysine-carrying linker (-Ahx-K-CONH_2_) was introduced to the C-end. A control peptide containing the same number of amino acids with nonsense sequence was also synthesized.

The three chimeras were engineered by separate chemical coupling of the peptides to anti-CR1 antibody as previously described [17,41]. Briefly, the monoclonal antibody and peptide solutions were mixed at a 20-fold molar excess of the peptides, and a zero-length crosslinking agent EDC (1-ethyl-3-(3′-dimethylaminopropyl) carbodiimide.HCl), (Sigma) was added for initiation of the coupling reaction at a 60-fold molar excess relative to the antibody. The ε-amino groups from the lysine residues of the peptide linker could interact with the available free carboxyl groups from the antibody structure. The engineered chimeras were as follows: a Tg1 chimera, a Tg2 chimera (containing the p949-969 or p1948-1968, respectively), and a Control chimera (containing the control peptide) were purified and concentrated.

### 4.6. Tg Peptide Epitopes Recognition and Anti-Tg Antibody Binding Inhibition

The thyroid gland lysate was tested for recognition by the anti-Tg antibodies in sera from the HT patients by ELISA. The lysate was diluted to 10 μg/mL in a coating buffer (NaHCO_3_, pH-9.6) and was then used for coating of immunoplates (Maxisorp, Nunc, Roskilde, Denmark) for 12h/4 °C. Next, the plates were washed with PBS/0.05% Tween 20 (T-PBS) and blocked with 1% bovine serum albumin (BSA) in T-PBS for 2 h at room temperature, followed by three washes. Serum samples from HT patients and healthy donors diluted 1:50 in T-PBS were added and incubated for 1h at RT followed by incubation with alkaline phosphatase-labeled goat anti-human IgG (Sigma-Aldrich). After washing, a phosphatase substrate (1 mg/mL pNPP, Sigma) was added and the absorbance was measured at 405 nm. The obtained results were presented as optical density (OD), corresponding to the titer of anti-Tg IgG antibodies.

Next, a competitive ELISA for epitope recognition was performed. Using the same experimental design, the sera from HT patients and healthy donors were preincubated with Tg1/2 peptide epitope mix (10 mM) or with the same quantity of control peptide for 1h/RT. The ELISA was then performed as described above.

### 4.7. Flow Cytometry Analysis

A competition binding assay for CD35 was performed using isolated PBMCs from HT patients. The cells (2 × 10^6^ cells/mL) were incubated with either Tg1, Tg2, the Control chimeras, with an unconjugated anti-CD35 MoAb (1 µg/10^6^ cells), or with PBS alone for 30 min at 4 °C, followed by two washes with a FACS buffer (PBS containing 2.5% FCS and 0.05% sodium azide). Subsequently, the lymphocytes were incubated with FITC-conjugated anti-human CD35 antibody combined with anti-human anti-CD19-eFluor450 or with anti-CD3 PE-Cy7 antibodies for 30 min at 4 °C.

In an independent experiment, PBMCs from HT patients were incubated with Tg1/Tg2 chimeras, with unconjugated anti-CD35 MoAb, or with PBS alone for 20 min at 4 °C, followed by incubation with anti-mouse IgG FITC antibody combined with anti-human anti-CD19-eFluor450 or with anti-CD3 PE-Cy7 antibodies for 30 min at 4 °C.

Next, after two washes, 50,000 cells from each sample were analyzed by a BD LSR II flow cytometer (BD Biosciences, Mountain View, CA) using Weasel v3.7.1. software (from Frank Battye).

### 4.8. ELISA for Tg-Epitope Recognition

To check the availability of Tg 1 and 2 epitopes in the composition of chimeric molecules for B-cell receptors, we performed an ELISA epitope recognition test. Tg1, Tg2, and the Control chimeras (10 µg/mL in coating buffer) were coated in 96-well immunoplates (Maxisorp, Nunc, Roskilde, Denmark) for 12 h at 4 °C. Further, the plates were blocked, washed, and incubated with pre-diluted (1:50 in T-PBS) sera from HT patients or healthy volunteers for 1 h at room temperature. Later, the ELISA development was performed as described above and the Tg1 and Tg2 peptide recognition was assessed spectrophotometrically.

### 4.9. Suppression of Proliferation Assay

PBMCs and TMCs from HT patients or PBMCs from healthy donors (2 × 10^6^ cells/mL) were incubated with increasing concentrations of the Tg 1/2 chimera mix, or with the Control chimera (8 ng/mL, 40 ng/mL, 200 ng/mL, 1000 ng/mL) in 96-well culture plates for 4 days. The control cells were stimulated with 50 ng/mL PMA (phorbol myristate acetate) plus 0.75 µg/mL Ionomycin, or cultured in a medium only. MTT was then added to the wells and the plates were incubated for further 4 h. The plates were centrifuged at 700 RPM for 10 min, the supernatant was aspirated, and dimethyl sulfoxide was added to each well. The absorbance of the dissolved formazan crystals was measured at 595 nm and the background subtracted at 620 nm.

### 4.10. ELISpot (Enzyme Linked Immunospot) Assay

To count the anti-Tg IgG antibody-secreting plasma cells we used the same experimental design as in the proliferation assay for the cultivation and treatment of PBMCs isolated from HT patients and healthy volunteers for 24 h. The cells were stimulated with 3 μg/mL CpG (InvivoGen, Toulouse, France) plus 5 μg/mL LPS (from *E. coli*, Sigma) for 3 additional days. The control samples were cultured in RPMI medium only. Later, the pre-incubated PBMCs were transferred to the respective wells in a 96-well ELISpot plate (Millipore, Bedford, MA, USA) pre-coated with 5 μg/mL Tg1/2 peptides (2.5 μg Tg1 + 2.5 μg Tg2) for 12–16 h at 4 °C and were further cultured for additional 4 h in a humidified 5% CO_2_ atmosphere at 37 °C. After extensive washing, the samples were incubated with an alkaline phosphatase-conjugated anti-human IgG antibody for 1 h and developed by NBT/BCIP substrate (Sigma). The number of colored spots corresponding to anti-Tg IgG antibody-producing plasmocytes was counted by C.T.L.Immunospot S6 Ultimate UV Image Analyzer (Bonn, Germany). Each donor (patient or healthy volunteer) was tested separately.

### 4.11. Apoptotic Assay

For apoptosis evaluation of PBMCs from HT patients and healthy individuals, the isolated cells (2 × 10^6^ cells/mL in complete RPMI-1640 mediums) were incubated for 2 days with increasing concentrations of the Tg1/2 chimeras or the Control chimera (8, 40, 200, 1000 ng/mL) at 37 °C/5% CO_2_. The untreated cells were used as controls. The collected cells were washed and stained with anti-human CD19-eFlour450 or anti-human CD3-PeCy7 antibodies together with Annexin V-FITC/PI Apoptosis kit (eBioscience™ Annexin V-FITC Apoptosis Detection Kit). Subsequently, the cells were washed and the apoptosis levels were evaluated within gated B- or T-cell populations by a BD LSR II flow cytometer counting 50,000 cells from each sample and were analyzed by Diva version 6.1.1. software (BD Biosciences, Mountain View, CA, USA).

### 4.12. Statistical Analysis

All statistical analyses were performed with GraphPad Prism 5 software (San Diego, CA). The one-way ANOVA test and the two-tailed Student’s *t*-test were used to determine the differences between each two groups. Values in the figures correspond to mean ± SD. A value of *p* < 0.05 was considered statistically significant.

## Figures and Tables

**Table 1:** Human Tg peptide fragments selected in the present study. The predicted binding cores are given in bold.

Position	Peptide	Binders	Covered HLA	Identity to Mouse Tg
949	SR**FPLGESFLVAKGIRLR**NE	8	DR3, DR4, DR5, DQ8	88%
1948	KA**LFRKKVILEDKVKNFY**TR	6	DR4, DR5, DQ2	75%

## Data Availability

The data presented in this study are available on request from the corresponding author. The data are not publicly available due to ethical reasons.
